# Supplementary material for: Identification of photoperiod-induced specific miRNAs in the adrenal glands of Sunite sheep (Ovis aries)
Source: Front Vet Sci. 2022 Jul 22;9:888207. doi: 10.3389/fvets.2022.888207 (PMC9354845; doi:10.3389/fvets.2022.888207)
Supplement: Supplementary file 1 [file Data_Sheet_1.ZIP › Supplementary file/miRDeep2 file.zip!/miRDeep2 file/Novel_Structure/LP42A1/Novel_297.pdf]

Provisional ID	:	NC_019459.2_4311
Score total	:	1.9
Score for star read(s)	:	-1.3
Score for read counts	:	0
Score for mfe	:	1.6
Score for randfold	:	1.6
Score for cons. seed	:	
Total read count	:	44
Mature read count	:	44
Loop read count	:	0
Star read count	:	0

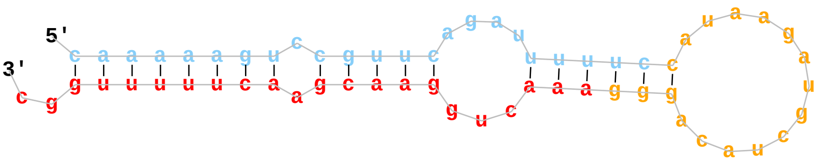

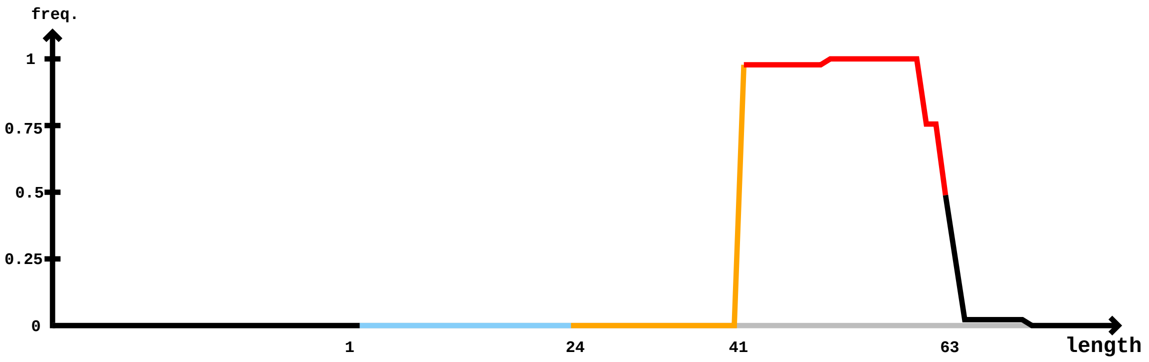

Star Mature

```
5'- ugcacgugugugucc guauauugg guugaacaaaaaguccguucagauuuuuccauaagaugcuacagggaaacuggaacgaacuuuuuggccaacccaauaauaauca   -3'    exp
```

```
5'- ugcacguguguguccguauauugg guugaacaaaaaguccguucagauuuuuccauaagaugcuacagggaaacuggaacgaacuuuuuggccaacccaauaauaauca   -3'    exp
    .((((....)))).....((((((((((..((((((((.(((((....((((((.............))))))...))))).))))))))..)))))))))).......    reads     mm     sample
    ......................................................................aaacuggaacgaacuuuuu....................    11        0      seq
    ......................................................................aaacuggaacgaacuuuuugg..................    12        0      seq
    ......................................................................aaacuggaacgaacuuuuuggc.................    21        0      seq
    ...............................................................................cgaacuuuuuggccaaccUaa.........    1         1      seq
```
